# Primates chunk simultaneously-presented memoranda

**DOI:** 10.3389/fnbeh.2022.1060193

**Published:** 2022-12-13

**Authors:** Charles D. Holmes, ShiNung Ching, Lawrence H. Snyder

**Affiliations:** ^1^Department of Biomedical Engineering, Washington University in St. Louis, St. Louis, MO, United States; ^2^Department of Neuroscience, Washington University in St. Louis, St. Louis, MO, United States; ^3^Department of Electrical Engineering, Washington University in St. Louis, St. Louis, MO, United States

**Keywords:** chunking, monkey, multi-item, primate, working memory

## Abstract

Though much research has characterized both the behavior and electrophysiology of spatial memory for single targets in non-human primates, we know much less about how multiple memoranda are handled. Multiple memoranda may interact in the brain, affecting the underlying representations. Mnemonic resources are famously limited, so items may compete for “space” in memory or may be encoded cooperatively or in a combined fashion. Understanding the mode of interaction will inform future neural studies. As a first step, we quantified interactions during a multi-item spatial memory task. Two monkeys were shown 1–4 target locations. After a delay, the targets reappeared with a novel target and the animal was rewarded for fixating the novel target. Targets could appear either all at once (simultaneous) or with intervening delays (sequential). We quantified the degree of interaction with memory rate correlations. We found that simultaneously presented targets were stored cooperatively while sequentially presented targets were stored independently. These findings demonstrate how interaction between concurrently memorized items depends on task context. Future studies of multi-item memory would be served by designing experiments to either control or measure the mode of this interaction.

## Introduction

A hallmark of animal behavior is the ability to integrate recent information to guide action and decision making. This ability is achieved with the memory systems of the brain, particularly working memory. Working memory is the cognitive construct underlying the active retention, manipulation, and utilization of information from either sensory systems or long-term memory. Working memory often involves retaining multiple pieces of information. Much remains unknown about how multiple memoranda are represented by prefrontal neurons. Individual representations may interact. Memory capacity is famously limited (Miller, 1956; Cowan, 2001; Luck and Vogel, 2013). Individual pieces of information are therefore likely to interfere with one another, as if competing for limited resources. Alternatively, pieces of information may be combined together for more efficient use of resources, either by intrinsic mechanisms or by active strategies such as “chunking,” effectively extending memory capacity and improving mnemonic performance (Brady et al., 2009; Zhang and Luck, 2011; Nassar et al., 2018). The characterization and, if possible, causal manipulation of competitive or cooperative interactions would facilitate the interpretation of the electrophysiology of multi-item memory.

**FIGURE 1:**
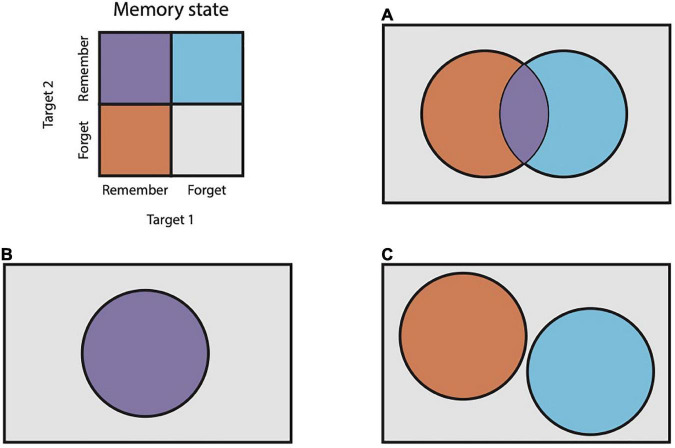
Venn diagrams of pairs of random variables. **(A)** Two random variables that are statistically independent. **(B)** Two random variables with positive correlation. **(C)** Two random variables with negative correlation. In this case, the variables are mutually exclusive.

Previous work supports the idea that animals other than humans may chunk memoranda together (Terrace, 1987; Tremblay et al., 2009; Scarf et al., 2018). In these studies, animals are trained to remember a sequence, e.g., a series of arm movements, and then report that sequence back in the same order that it was presented. Experimenters find that execution times of elements of the reported sequence are temporally clustered and reason that this clustering reflects an internal process of chunking. We wished to obtain more direct evidence for chunking, i.e., for cooperative interactions between memoranda. To accomplish this, we reasoned that mnemonic interactions can be quantified as the degree of dependence between the memory state probabilities of multiple items during a memory task. The memory of each item can be treated as a random variable with one of two outcomes: “remembered” or “forgotten.” The dependence between two such variables can be captured with measures of correlation. If two memories are stored independently, then the probabilities of remembering or forgetting each item should be statistically independent (Figure 1A). In this case, correlation will approach zero. If the two memories are stored cooperatively, then they should be remembered or forgotten in concert more often than not, resulting in positive correlation (Figure 1B). If the two memories compete for limited resources, their recall should approach mutual exclusion, resulting in a negative correlation (Figure 1C).

We applied this logic to monkeys remembering 1–4 targets at once. These targets could appear either simultaneously or sequentially with a delay between them. After the delay, the animals were presented with all targets at once along with a novel target. Animals were rewarded for making a saccade to the novel target, encouraging them to remember each of the memory targets. Additionally, the design facilitates neuronal recording studies by reducing the chances of confounding motor planning with memory *per se*. We hypothesized that simultaneously presented targets would be more likely encoded in a combined fashion while sequentially presented targets would be more likely encoded independently or even in competition with one another. We found that sequentially presented items are stored independently, i.e., remembering one target is uncorrelated with remembering another target. In contrast, simultaneously presented items showed cooperativity or chunking—remembering one target means that it is more likely that the other target is remembered. These findings have implications for the interpretation of neuronal activity during multi-item memory as well as for the design of memory tasks that investigate multi-item memory.

## Materials and methods

### Experimental design

Two male rhesus macaques (*Macaca mulatta*) participated in the study. All procedures conformed to the Guide for the Care and Use of Laboratory Animals and were approved by the Washington University Institutional Animal Care and Use Committee.

Each animal was fit with a head post (Graymatter Research, MT, USA). During each experiment, the animal sat in a customized primate chair (Crist Instrument, MD, USA) within a completely dark room. Visual stimuli were either back projected onto a screen in front of the animal using a cathode ray tube projector (Nippon Electric Company, Japan) or shown on an organic light-emitting-diode television set (LG, South Korea). Each method has a very high dynamic range so that there was no extraneous light to provide cues other than the fixation point during the delay intervals. Eye positions were monitored with an infrared video eye-tracking system (ISCAN, MA, USA).

### Behavioral task

**FIGURE 2:**
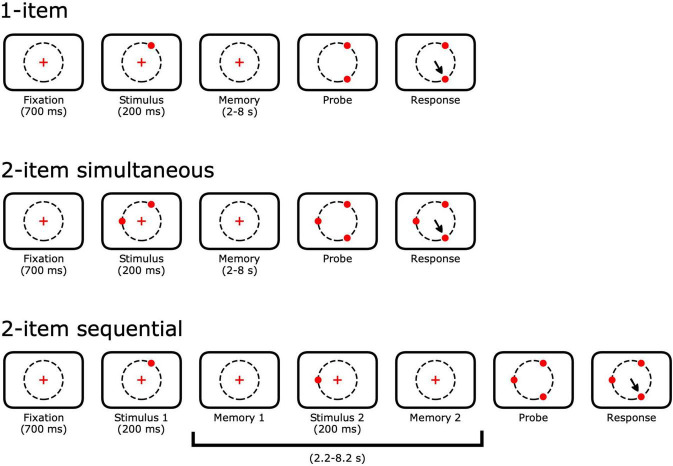
Single- and multi-item spatial memory tasks. Trials begin with a 700 ms fixation of a central point. For *n*-item simultaneous trials, *n* peripheral targets appear 13 degrees of visual angle (dva) out from the fixation point. Animals continue to fixate through a variable delay period. For *n*-item sequential trials, a single target appears first and subsequent targets appear throughout the full delay. For all trial types, all of the previously shown peripheral targets reappeared at the end of the delay along with a single novel target. Animals received liquid rewards for making a saccade to the novel target. Delay period lengths were varied across sessions and ranged from 2 to 8 s.

Animals performed a spatial memory task with up to 4 spatial memoranda (targets) presented either simultaneously or sequentially. Each trial began with the animal gazing within 3 degrees of visual angle (dva) of a central fixation point for 700 ms (Figure 2). Peripheral memory targets appeared for 150 or 200 ms. The animal continued to fixate through a delay. After the delay, the fixation point disappeared and all of the memory targets reappeared along with one novel peripheral target. The animal was given a liquid reward for shifting his gaze to the novel target within 150 ms of leaving the fixation point. Throughout the task, the animal was permitted to blink without aborting the trial. Blinks were defined as periods when the pupil was at least 20% occluded. Eye positions during blinks were replaced with interpolation based on the eye positions before and after the blink.

Trial outcomes for our memory task are ambiguous: it is unclear if the animal remembers every memory target or just a subset of the targets and guesses correctly. An alternative methodology would be for the animal to make a series of saccades, one to each memory target. However, we designed this task to be used during electrophysiological experiments. Were the animal to make a series of saccades to each target, the memorized target location and the saccade plan would be confounded. For example, it would be ambiguous whether neuronal activity related to the target of the second saccade would represent that target’s location relative to the fixation point or relative to the target of the first saccade. Instead, by having the animal saccade to a novel target at the end of the trial, the animal saccade plan consists of only one movement from the fixation point and is thus unambiguous. Additionally, the animal is unable to plan the saccade until the end of the trial. Thus, activity during the delay is more likely to represent memory *per se* rather than a saccade plan.

Delay duration varied over 2–8 s. The duration was set by the experimenter, based on the animal’s performance history, with the goal of maximizing both the delay and the animal’s performance. Within a block, delay only varied by up to 500 ms. Target presentation duration was invariant within a session, lasting 150 ms in earlier sessions and 200 ms in later sessions.

Memory targets appeared either simultaneously or sequentially. During simultaneous trials, every memory target appeared and disappeared at the same time before the delay. During sequential trials, targets were presented one at a time. The first memory target appeared and disappeared before the delay. During 2-item trials, the delay was interrupted after 33–67% (randomized trial-to-trial) of its duration. The second memory target appeared and disappeared, the delay resumed, and the trial continued as in simultaneous trials. During 3-items trials, the second and third targets were shown after 33 and 67% of the delay, respectively. During 4-item trials, the second, third, and fourth targets appeared after 25, 50, and 75% of the delay, respectively.

Targets appeared on an invisible circle centered on the fixation point with a radius of 13 dva. In 1-item trials, the memory target location was randomized in 1 degree of arc (deg) steps, for a total of 360 possible locations. In 2-item trials, the second memory target appeared ± 60, ± 120, or 180 deg from the first target, for a total of 1,800 (360 × 5) possible target configurations. In 3- and 4-item trials, targets were spaced by multiples of 15 deg, yielding 182,160 (360 × 23 × 22) and 3,825,360 (360 × 23 × 22 × 21) possible target configurations, respectively. The novel target followed the same spacing constraints as memory targets.

Trials were aborted if the animal broke fixation. Trials were also aborted if the animal did not make a saccade within 2 s of the go cue. To encourage good performance, the animal was only given a liquid reward if a saccade landed within 4.5 dva of the novel target within 150 ms of leaving the fixation point.

During nearly every session, animals performed 1- and 2-item trials, both simultaneous and sequential. For a subset of sessions, 3- and/or 4-item trials, both simultaneous and sequential, were included and interleaved. Additionally, for some sessions, some 2-item trials were modified to omit one of the memory targets during the response phase (see section “Results”).

### Behavioral model

We modeled the animal’s memory state during 2-item trials as a bivariate Bernoulli random variable, [*M*_1_,*M*_2_]. The two dimensions of this variable correspond to the two memory targets. Each dimension can take on a value of one or zero, with one representing the target that was remembered and zero representing the target that was forgotten.

We quantify the level of association between *M_1_* and *M_2_* with correlation, computed as:


$$\begin{array}{l} R=\\ \frac{P(M_{1}=1,M_{2}=1)P(M_{1}=0,M_{2}=0)-P(M_{1}=0,M_{2}=1)P(M_{1}=1,M_{2}=0)}{\sqrt{P(M_{1}=1)P(M_{1}=0)P(M_{2}=1)P(M_{2}=0)}} \end{array}$$

We modeled the animals’ behavior (choices) as a random variable, *C*, with three possible outcomes: the novel target is chosen (*c_n_*), the first memory target is chosen (*c_1_*), or the second memory target is chosen (*c_2_*). We assume that the animal will choose from the set of targets he does not remember, i.e., the novel target and any forgotten target, and that selection will be unbiased, i.e., uniformly random. For example, if he remembers the first target and forgets the second target, he will choose the novel target 50% of the time and the second target the other 50% of the time. The relationship between the animal’s choice frequencies and memory state probabilities is expressed as:


$$\begin{array}{l} \left[\begin{array}{c} P(C=c_{n})\\ \begin{array}{l} P(C=c_{1})\\ P(C=c_{2}) \end{array} \end{array}\right]\\ =\left[\begin{array}{l} \begin{array}{cccc} 1 & 1/2 & 1/2 & 1/3 \end{array}\\ \begin{array}{cccc} 0 & \;0 & \;1/2 & 1/3 \end{array}\\ \begin{array}{cccc} 0 & 1/2 & \;0 & \;1/3 \end{array} \end{array}\right]\;\left[\begin{array}{l} \begin{array}{c} P(M_{1}=1,M_{2}=1)\\ P(M_{1}=1,M_{2}=0) \end{array}\\ \begin{array}{c} P(M_{1}=0,M_{2}=1)\\ P(M_{1}=0,M_{2}=0) \end{array} \end{array}\right] \end{array}$$

During simultaneous trials, there is no meaningful distinction between the first and second memory target. In this case, we assume that the probability of remembering only the first target is identical to the probability of remembering only the second target. We thus simplify our system of equations to be:


$$\begin{array}{l} \left[\begin{array}{c} P(C=c_{n})\\ P(C=c_{1})+P(C=c_{2}) \end{array}\right]=\left[\begin{array}{l} \begin{array}{ccc} 1 & 1/2 & 1/3 \end{array}\\ \begin{array}{ccc} 0 & 1/2 & 2/3 \end{array} \end{array}\right]\\ \left[\begin{array}{c} P(M_{1}=1,M_{2}=1)\\ P(M_{1}=1,M_{2}=0)+P(M_{1}=0,M_{2}=1)\\ P(M_{1}=0,M_{2}=0) \end{array}\right] \end{array}$$

The aforementioned systems are underdetermined: simultaneous trials have 2 known parameters and 3 unknown parameters; sequential trials have 3 known parameters and 4 unknown parameters. As a consequence, there is no single solution in either case. However, because probabilities are bounded between zero and one, the space of solutions is bounded. We report the solutions that minimize and maximize the probability of forgetting both targets.

For a subset of sessions, the animals performed a variant of 2-item trials in which one memory target was omitted at the end of the trial. We distinguish these trial variants (omission trials) from the standard trials via two parameters, *O_1_* and *O_2_*, where *O*_*x*_ = 1 if target *x* was omitted and *O*_*x*_ = 0 otherwise. Thus, for sequential trials, the system of equations becomes:


$$\begin{array}{l} \left[\begin{array}{l} \begin{array}{c} P(C=c_{n}|O_{1}=0,O_{2}=0)\\ P(C=c_{1}|O_{1}=0,O_{2}=0) \end{array}\\ P(C=c_{2}|O_{1}=0,O_{2}=0)\\ P(C=c_{n}|O_{1}=0,O_{2}=1)\\ P(C=c_{1}|O_{1}=0,O_{2}=1)\\ P(C=c_{n}|O_{1}=1,O_{2}=0)\\ P(C=c_{2}|O_{1}=1,O_{2}=0) \end{array}\right]\\ =\left[\begin{array}{l} \begin{array}{cccc} 1 & 1/2 & 1/2 & 1/3 \end{array}\\ \begin{array}{cccc} 0 & \;0 & \;1/2 & 1/3 \end{array}\\ \begin{array}{cccc} 0 & 1/2 & \;0 & \;1/3 \end{array}\\ \begin{array}{cccc} 1 & \;1 & \;1/2 & 1/2 \end{array}\\ \begin{array}{cccc} 0 & \;0 & \;1/2 & 1/2 \end{array}\\ \begin{array}{cccc} 1 & 1/2 & \;1 & \;1/2 \end{array}\\ \begin{array}{cccc} 0 & 1/2 & \;0 & \;1/2 \end{array} \end{array}\right]\;\left[\begin{array}{l} \begin{array}{c} P(M_{1}=1,M_{2}=1)\\ P(M_{1}=1,M_{2}=0) \end{array}\\ \begin{array}{c} P(M_{1}=0,M_{2}=1)\\ P(M_{1}=0,M_{2}=0) \end{array} \end{array}\right] \end{array}$$

For simultaneous trials, the system of equations becomes:


$$\begin{array}{l} \left[\begin{array}{c} P(C=c_{n}|O_{1}=0,O_{2}=0)\\ \begin{array}{l} P(C=c_{1}|O_{1}=0,O_{2}=0)+P(C=c_{2}|O_{1}=0,O_{2}=0)\\ \begin{array}{c} P(C=c_{n}|O_{1}=0,O_{2}=1)+P(C=c_{n}|O_{1}=1,O_{2}=0)\\ P(C=c_{1}|O_{1}=0,O_{2}=1)+P(C=c_{2}|O_{1}=1,O_{2}=0) \end{array} \end{array} \end{array}\right]\\ =\left[\begin{array}{l} \begin{array}{ccc} 1 & 1/2 & 1/3 \end{array}\\ \begin{array}{ccc} 0 & 1/2 & 1/3 \end{array}\\ \begin{array}{ccc} 1 & 3/4 & \;1 \end{array}\\ \begin{array}{ccc} 0 & 1/2 & \;1 \end{array} \end{array}\right]\;\left[\begin{array}{l} \begin{array}{c} P(M_{1}=1,M_{2}=1)\\ P(M_{1}=1,M_{2}=0) \end{array}\\ \begin{array}{c} P(M_{1}=0,M_{2}=1)\\ P(M_{1}=0,M_{2}=0) \end{array} \end{array}\right] \end{array}$$

These systems of equations assume the probabilities of remembering targets are identical across trial variants, but in practice the animal’s behavior is noisy which can cause a solution probability to fall outside [0,1]. To correct for this, we solved the system of equations with a gradient descent algorithm while constraining values to [0,1].

### Confidence intervals

For many statistics, we estimate confidence intervals. For hit rates, we compute confidence intervals analytically. For correlations of memory probabilities, we use simple bootstrapping; trial blocks, not individual trials, are resampled with replacement and the statistic under consideration is recomputed. This procedure is repeated 1000 times and the 2.5th and 97.5th percentile are taken as the bounds of a 95% confidence interval.

## Results

### Behavior

Two male rhesus macaques (*Macaca mulatta*; F and H) remembered the spatial locations of up to four targets (items) for 2–8 s (Figure 2). At the end of each trial, the central fixation point disappeared and the memory targets reappeared along with a novel target, cuing the animal to make a saccade. The animal was rewarded for transferring fixation to the novel target. If the animal failed to fixate the novel target within 150 ms of leaving the central fixation point, the trial ended with no reward. This time constraint meant that the animals could not “search” for the target that would give them a reward, but had to move directly to the target in a single saccade or, rarely, one saccade plus an immediate and quick corrective saccade.

Monkeys F and H participated in 40 and 79 experimental sessions, respectively, and completed an average of 730 ± 400 (mean ± standard deviation) and 630 ± 370 trials per session. During nearly every session, animals performed 1-item, 2-item simultaneous, and 2-item sequential trials (Tables 1, 2). In a subset of sessions, animals also performed 3- and 4-item trial variants. All trial types performed in a given session were interleaved. Delay durations were adjusted within session to maximize delay length while maintaining a hit rate (across all trial types) above 70%. Delay lengths of all trial types were kept the same within each block of trials so that hit rates could be directly compared.

**TABLE 1:** Number of sessions in which Monkey F performed each trial type.

Timing	Load	Sessions
*n/a*	1	40 (100%)
Simultaneous	2	40 (100%)
	3	17 (43%)
	4	16 (40%)
Sequential	2	40 (100%)
	3	17 (43%)
	4	16 (40%)

**TABLE 2:** Number of sessions in which Monkey H performed each trial type.

Timing	Load	Sessions
*n/a*	1	79 (100%)
Simultaneous	2	79 (100%)
	3	41 (52%)
	4	39 (49%)
Sequential	2	78 (99%)
	3	38 (48%)
	4	37 (47%)

**FIGURE 3:**
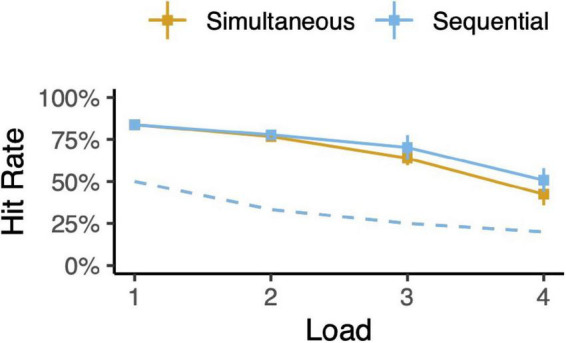
Task performance. Hit rates for each trial type (simultaneous or sequential) as a function of memory load. Error bars depict 95% confidence intervals. Dotted lines indicate chance levels.

The animals’ behavior indicated that they understood the task. Saccades were directed to within 4.5 dva of one of the available targets in almost every trial. The animals failed to leave the fixation point in 1% of trials. Regardless of the number of memory targets (load), the animals chose the novel target significantly more often than chance (Figure 3 and Supplementary Figure 1; binomial tests, all *p* < 10^–6^, no multiple-comparisons corrections). This was also true for each and every spatial arrangement of targets for 1- and 2-item trial types (Supplementary Figure 2; all *p* < 10^–6^). Performance varied with delay duration. For each 1- and 2-item trial type and delay length, hit rates were greater than chance (Supplementary Figure 3; all *p* < 10^–6^), though hit rate declines with delay (all slopes < 0, all *p* < 0.01). These results also held when we considered each monkey’s behavior individually.

### Simultaneously presented memory targets

We aimed to measure the association between simultaneously presented memory targets, focusing on the simplest case: two memory targets. To accomplish this, we compute a correlation value for two binary variables representing whether each memory target was remembered or forgotten (see section “Materials and methods”). Estimating these variables is non-trivial as the variables are not directly observable. However, probabilities can be inferred from the animal’s behavior.

We model behavior during simultaneous trials as follows: There are three possible memory states for each trial: (1) both targets are remembered, (2) only one target is remembered while the other is forgotten, and (3) both targets are forgotten. Critically, none of these three memory states map in a one-to-one manner with a particular behavioral outcome, i.e., whether or not the animal chooses the novel target. For example, if on a given trial the animal correctly chooses the novel target, it could mean that he remembered both memory targets. Alternatively, the animal may have forgotten one or both memory targets and arrived at the correct answer only by chance. We specify a system of equations relating the frequency of each choice to the probability of each memory state. Choice frequencies are then expressed as the sum of each memory state’s probability weighted by a guess rate (see section “Materials and methods”). Memory state probabilities can be estimated by solving this model. However, the model is underdetermined—the number of unknown parameters (the probabilities of the three memory states) exceeds the number of known parameters (the frequencies of the two possible behavioral outcomes). As a result, a bounded continuum of solutions exists. This range may or may not be consistent with one particular mode of association.

**FIGURE 4:**
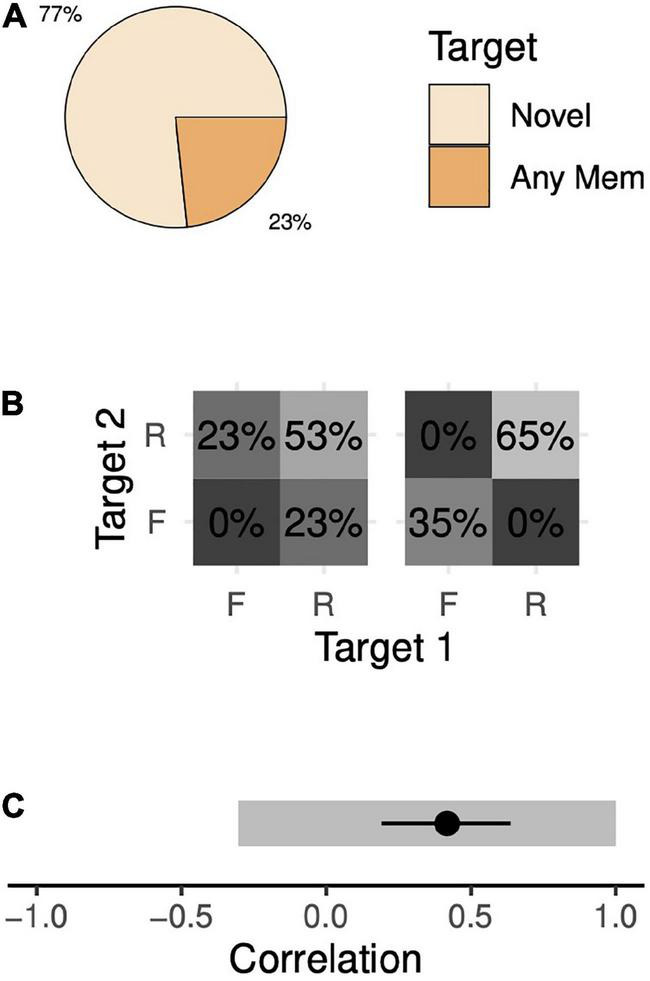
Target choice probabilities and memory probabilities during the 2-item simultaneous task. **(A)** The trial-wise probabilities of the animal choosing either the novel target or a memory target (either target 1 or 2) given that the animal chose a target (i.e., the animal moved his eyes to within 4.5 dva of either the novel target or a memory target). The animals rarely failed to choose a target after leaving the fixation point (4% of trials, see Supplementary Figure 4). **(B)** We assume a model in which the probabilities of choosing targets depend on the probabilities of remembering the targets (see section “Materials and methods”). We estimated a range of solutions, varying an assumed value for the probability of forgetting both targets. Here, we show two extreme solutions—when the probability of forgetting both targets is minimal (left), i.e., zero, and when the probability of forgetting both targets is maximal (right). R, remembered; F, forgotten. Note that these values do not map in a one-to-one fashion with the values in panel **(A)**. **(C)** Estimated correlation values between the memory of the two targets. Gray segments indicate the range-estimates of possible correlation coefficient values estimated with standard trials. With the addition of an additional trial type (omission trials, see section “Results”), we are able to make point estimates of correlation. The black circle and line indicate a point-estimate mean and 95% confidence interval, respectively.

During simultaneous 2-item trials, the animals correctly chose the novel target 77% of the time and incorrectly chose one of the memory targets 23% of the time (Figure 4A). Because there is no meaningful distinction between the two memory targets, we assume their frequencies of being chosen are equal (12%, each). A wide range of memory state probabilities are consistent with these data. At one extreme, the animals could remember both targets 53% of the time and remember just one target (but not the other) 47% of the time (Figure 4B, left). The animal would choose the novel target whenever he remembers both targets (100% of 53% of trials) and in half of the trials when he remembers only one target (50% of 47% of trials). This sums to 77% of trials and thus is consistent with our observations. This model implies a competitive interaction (correlation of –0.30; 95% confidence interval [CI], [–0.32, –0.29]). At the other extreme, the animal could remember both targets 65% of the time, forget both targets 35% of the time and never remember just one target (Figure 4B, right). In this case, the animal would choose the novel target in every trial when he remembers both targets (100% of 65%) and in one-third of those trials when he forgets both targets (33% of 35%) for a total of 77% of trials. This alternative model is also consistent with our observations, but, unlike the previous model, implies a cooperative interaction (correlation of +1.0; 95% CI, [+1.0, +1.0]). In between these two extremes lie a continuum of other memory models, each consistent with our observations, with correlations ranging from –0.30 to +1.0 (Figure 4C).

This wide range of consistent models is not due to noise. Rather, it is due to the underdetermined nature of the model. Thus, collecting arbitrarily many trials will not narrow the range. Instead, to remedy this issue, we collected data from the animals with a variant of simultaneous 2-item trials in which one of the memory targets was omitted at the end of the trial (omission trials, see section “Materials and methods”). Both animals performed the task with high hit rates after only a few trials, further supporting that they understood the task. By interleaving these omission trials with our standard trials, we increase our number of known parameters from 2 to 4 (2 behavioral outcomes x 2 trial types) while leaving the number of unknown parameters unchanged. The model is no longer underdetermined. Solving this extended system of equations yielded a positive degree of association (Figure 4C; R = +0.42, 95% CI [+0.19, +0.64]). Each animal’s data supported this conclusion when considered separately (Monkey H, R = +0.55, 95% CI [+0.28, +0.79]; Monkey F, R = +0.19, 95% CI [–0.21, +0.53]). Thus, the animals’ behavior with simultaneous presentations is consistent with a moderate degree of positive correlation, or chunking.

### Sequentially presented memory targets

We next determined the association between sequentially presented targets. Unlike simultaneous trials, sequential trials include an asymmetry between the memory targets that must be accounted for in the model. Thus, instead of three possible memory states, there are four: (1) both targets are remembered, (2) both targets are forgotten, (3) the first target is remembered while the second is forgotten, and (4) the second target is remembered while the first is forgotten. Additionally, there are three possible behavioral outcomes, one per target present during the response phase.

**FIGURE 5:**
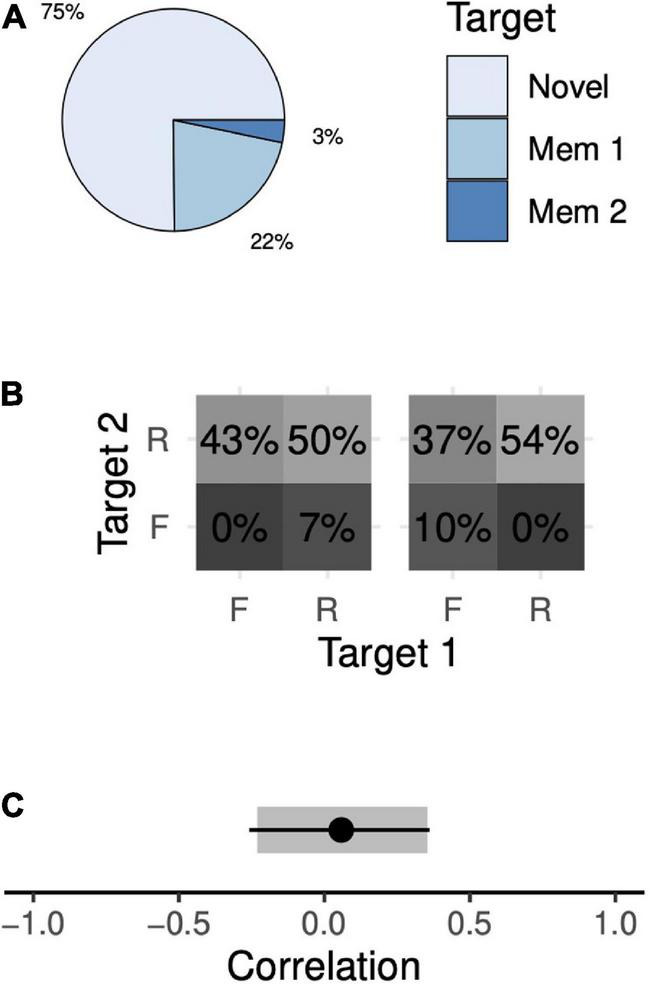
Target choice probabilities and memory probabilities during the 2-item sequential task. Format identical to Figure 4.

The animals chose the novel target in 75% of 2-item sequential trials, the first memory target in 22% of trials, and the second memory target in 3% of trials (Figure 5A). Only a narrow range of memory probabilities are consistent with these data. Across all solutions, the animal remembers both memory targets with a probability of 50–54%, only the first target in 0–7% of trials, only the second target in 37–43% of trials, and neither target in 0–10% of trials (Figure 5B). These data demonstrate a recency effect: the animal chose the first target more often than the second, suggesting that the second, more recent target was preferentially remembered. Once again, there was a wide range of models consistent with our observations, with correlations ranging from –0.23 (95% CI, [–0.25, –0.21]) to +0.35 (95% CI, [+0.32, +0.39]; Figure 5C).

As with simultaneous trials, we trained the animals to perform a sequential 2-item trial variant in which one of the memory targets was omitted. On a given trial, either the first or the second target could be omitted. The animals chose the novel target at rates far above chance (96 and 78% of trials when the first and second memory targets were omitted, respectively; both values greater than chance, *p* < 10^–6^). This manipulation allowed us to compute a point estimate of correlation. The animals’ behavior exhibited a small positive correlation that was not significantly different from zero (Figure 5C, *R* = +0.05, 95% CI, [–0.25, +0.36]). When considered separately, each animal’s data were consistent with this result (Monkey F, *R* = –0.16, 95% CI, [–0.22, +0.36]; Monkey H, *R* = +0.11, 95% CI, [–0.28, +0.37]). Thus, with sequential presentations, the animals’ behavior is consistent with independent storage.

## Discussion

To date, little is known about the neuronal basis of multi-item working memory. One complicating factor for such studies is that multiple items may not be stored independently. Individual pieces of information may interact in memory, either by competing for resources or by combining in a cooperative fashion. The manner in which items interact may vary depending on task demands and the subject’s strategy, e.g., chunking. Indeed, some studies have suggested that monkeys can chunk multiple memoranda (Terrace, 2002; Tremblay et al., 2009; Scarf et al., 2018). Generally, these studies have animals perform sequential motor responses and assume that relatively short intervals separate movements (i.e., memories) in a chunked sequence while relatively long intervals separate one chunked sequence from another. When animals must respond by moving to each memorandum in turn, subjects may remember a sequence of pre-planned responses rather than a set of spatial locations. This adds interpretational complexity by opening the possibility of different coding schemes. For example, if the task is to move to the remembered locations in a sequence, then the animal may remember the second location with reference to the endpoint of the first response, e.g., when presented with a target up and to the left followed by a target on the right, the animal may plan an up and to the left movement followed by a down and to the right movement. A related issue is that an animal may remember a path rather than a sequence of items, thereby biasing the animal to chunk the items. Furthermore, temporal chunking in the response sequence may reflect a property of movement execution rather than of the memory itself.

We take a more direct approach to examine chunking and other modes of association by explicitly examining the probabilistic association between memorized pieces of information. Our animals remembered up to four spatial locations at once, then identified a novel target among the original targets. For analytic simplicity, we focus on 2-item trials. Association is quantified with correlation between the memory of each of the two targets, with positive correlations indicating cooperative storage, i.e., memoranda tend to be remembered or forgotten together, and negative correlations indicating competitive storage, i.e., memoranda tend to be remembered when the other memorandum is forgotten and vice versa. A correlation of zero indicates independent storage. We found that simultaneous target presentation was associated with moderate chunking while sequential presentation was associated with independence.

Our animals’ behavior suggests that they knew to make a choice between targets on each trial. In only 1% of trials did they fail to leave the fixation point. Furthermore, if they left the fixation point, they moved their eyes to with 4.5 dva of a peripheral target in 96% of trials (Supplementary Figure 4). Our behavioral model assumes that the animals will always choose the novel target if they remember both memory targets but may erroneously choose a memory target they have forgotten. Our model does not consider trials in which the animal failed to make a choice; however, because these trials constitute such a small percentage of the total, we reasoned they can be excluded from analyses (with the exception of Supplementary Figure 4).

As expected, memory performance decreased with memory load (Figure 3 and Supplementary Figure 1). This trend is hypothesized to be a driving factor of chunking; chunking combats limited memory capacity by effectively compressing information (Brady et al., 2009; Zhang and Luck, 2011; Nassar et al., 2018). For loads above 2, there is a suggestion that simultaneous trials are harder for the animal than sequential trials—sequential trial hit rates are greater than simultaneous trial hit rates. Given that we report chunking in simultaneous trials but not sequential trials, one may have hypothesized that simultaneous trials should be easier than sequential trials and thus simultaneous trial hit rates should be higher. However, sequential trials may be inherently easier than simultaneous trials. For example, the animal need only remember the second target for a fraction of the full delay. As another example, the animal may have fewer encoding errors when targets are presented one-at-a-time as compared to all at once. In any case, we do not make any claims about relative difficulty of simultaneous versus sequential trials, with or without chunking.

Our approach avoids the confounds of sequential motor planning, but unfortunately contains a critical flaw: our memory task provides only 1 fewer known measurement (behavioral outcomes) than unknown measurement (possible memory states). Thus, a model based only on data from these tasks is underdetermined (Figures 4, 5). We further constrained the model by adding a trial variant in which just one memory target and one novel target were presented at the end of the trial (omission trials). This allows us to make a point estimate of correlation and reveals that associations are cooperative with simultaneous target presentations and independent with sequential presentations.

The mode of association has implications on the interpretation of electrophysiological data. During memory tasks with simultaneously- and sequentially presented targets, one may expect to observe correlates of cooperative and independent storage, respectively, during memory. For example, cooperative storage may manifest as individual neurons representing multiple targets at once. Furthermore, the representation of multiple cells may be more complicated than simply the sum of each target’s 1-item trial representation. In contrast, independent storage may manifest as individual cells encoding one and only one target at a time.

One limitation of our methodology is the assumption that errors reflect a failure of memory rather than a failure of encoding. This assumption may lead to a potential confound: If on a subset of trials the animal was disengaged and did not encode both targets during stimulus presentation, estimates of the proportion of trials when the animal forgets both targets would be artificially increased. In turn, estimates of correlation would also be artificially increased. However, delay intervals are many seconds long. With long trials, the best strategy after failing to encode the targets is to terminate the trial early by breaking fixation rather than continuing the trial and settling for the 33% reward rate produced by guessing. If the aim is to maximize overall rewards per unit time, then aborting the trial early is the optimal strategy. If they were so disengaged as to not care about the reward rate for that trial, then they would have no reason to make any saccade at all—yet both animals made saccades to peripheral targets on 99.0 and 99.6% of trials, respectively.

Competitive interactions may have arisen if coding one target distracts the animal from coding the second target. One can imagine this happening with simultaneous target presentations if animals have time to attend to only one of the two targets during the brief presentation interval, but we observed positive rather than negative correlations for simultaneous presentations. In a sequential task, one can imagine that encoding the first target might produce something akin to an attention blink (for a review, see Dux and Marois, 2009), which might then interfere with the encoding of the second target. This would result in competitive interactions in the sequential task as well as a primacy effect (the first target would be remembered more often than the second). Instead, we observe no correlation (independent storage) and a recency effect.

One can also imagine that animals choose not to encode the first target of a sequential trial, forgoing a potential 100% reward rate for remembering both targets and instead accepting a 50% reward rate for the much easier task of remembering just a single target. We can rule this out, however, by observing that, until the second item appears, the 2-item sequential task is identical to the 1-item task. Both animals perform very well in 1-item trials, indicating that they are in fact encoding the first item that appears in a trial.

With closely spaced targets, it is conceivable that the second target could exert a backward masking effect (for a review, see Breitmeyer, 2007) on the first target and thereby produce a recency effect that depends on encoding. This is unlikely, however, since the shortest inter-target interval that we used was 2 s, which is longer than most reports of backward masking. Thus, we believe that the effects we observe are likely to reflect interactions that occur during the memory period or memory read-out (retrieval) rather than interactions that occur during encoding.

## Data availability statement

The raw data supporting the conclusions of this article will be made available by the authors, without undue reservation.

## Ethics statement

This animal study was reviewed and approved by the Washington University Institutional Animal Care and Use Committee.

## Author contributions

CH designed, conducted the experiment, analyzed the data, and wrote the manuscript. SC and LS helped design the experiment and write the manuscript. All authors contributed to the article and approved the submitted version.
