# Supplementary material for: Primates chunk simultaneously-presented memoranda
Source: Front Behav Neurosci. 2022 Dec 13;16:1060193. doi: 10.3389/fnbeh.2022.1060193 (PMC9792603; doi:10.3389/fnbeh.2022.1060193)
Supplement: Supplementary file 1 [file Data_Sheet_1.PDF]

Supplementary Figures

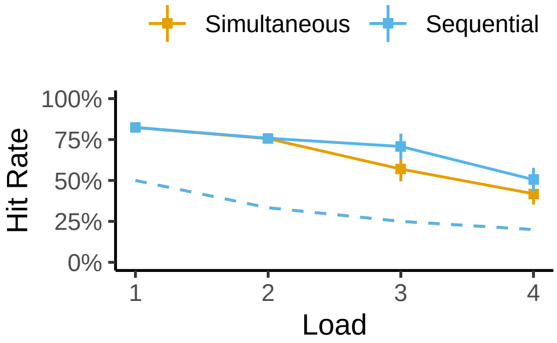

Figure 1: Related to Figure 3. Format and analysis are identical to Figure 3 with one exception — only trial blocks with 1-, 2-, 3-, and 4-item trials are included (Simultaneous, n = 55, 16 from Monkey F, 39 from Monkey H; Sequential, n = 53, 16 from Monkey F, 37 from Monkey H).

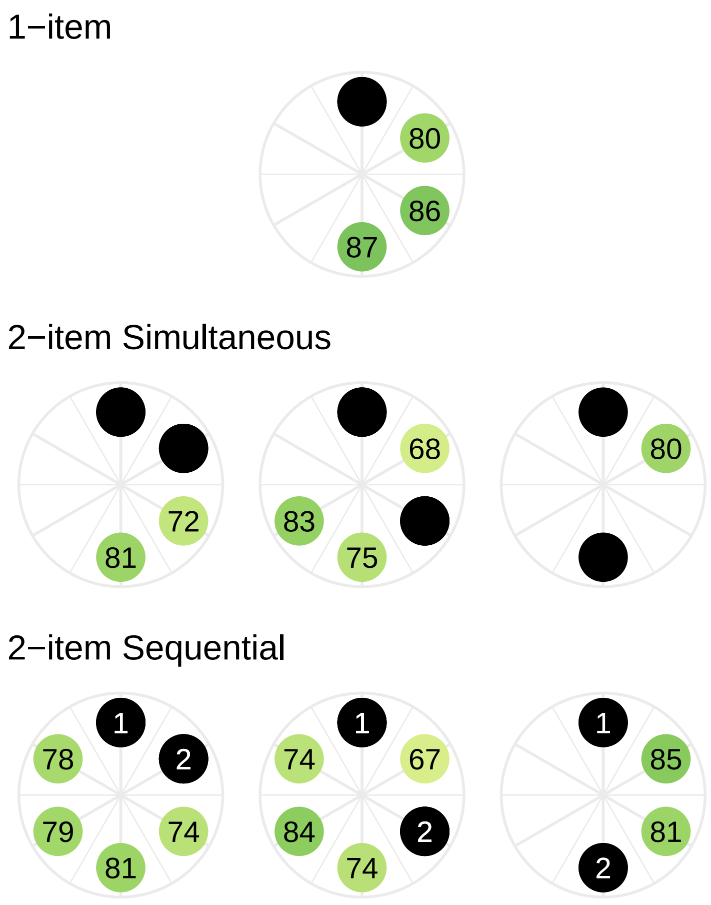

Figure 2: Hit rates for each 1- and 2-item trial type and spatial arrangement of targets.

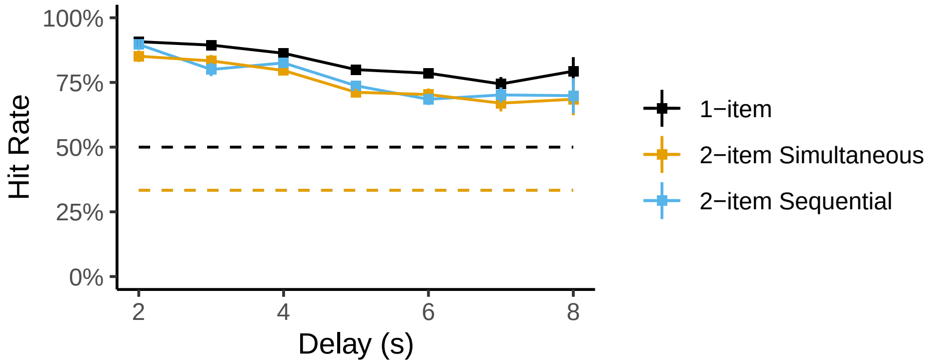

Figure 3: Hit rates for each 1- and 2-item trial type and delay duration rounded to the nearest s. Error bars depict 95% confidence intervals. Dotted lines indicate chance levels.

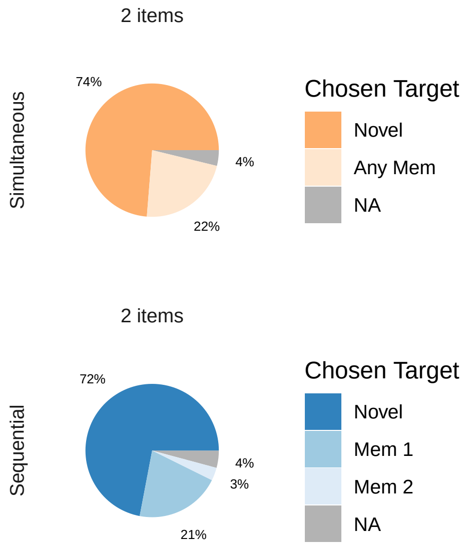

Figure 4: Related to Figures 4 and 5. The trial-wise probabilities of the animal choosing, the novel target, choosing a memory target, or failing to choose any target (NA) given that the animal's eyes left the fixation point after the go-cue.
